# Supplementary material for: Identification of Freezing-Responsive microRNAs and Their Targets in Chinese Jujube by Small RNA and Degradome Sequencing
Source: Int J Mol Sci. 2026 Sep 4;27(17):7912. doi: 10.3390/ijms27177912 (PMC13566890; doi:10.3390/ijms27177912)
Supplement: Supplementary file 1 [file ijms-27-07912-s001.zip › Supplementary Material S2/novel_miR_88.pdf]

Provisional ID	:	novel_miR_88
Score total	:	9280
Score for star read(s)	:	3.9
Score for read counts	:	9271.8
Score for mfe	:	2.6
Score for randfold	:	1.6
Score for cons. seed	:	
Total read count	:	18198
Mature read count	:	18148
Loop read count	:	0
Star read count	:	50

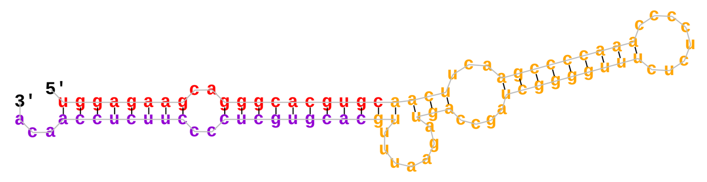

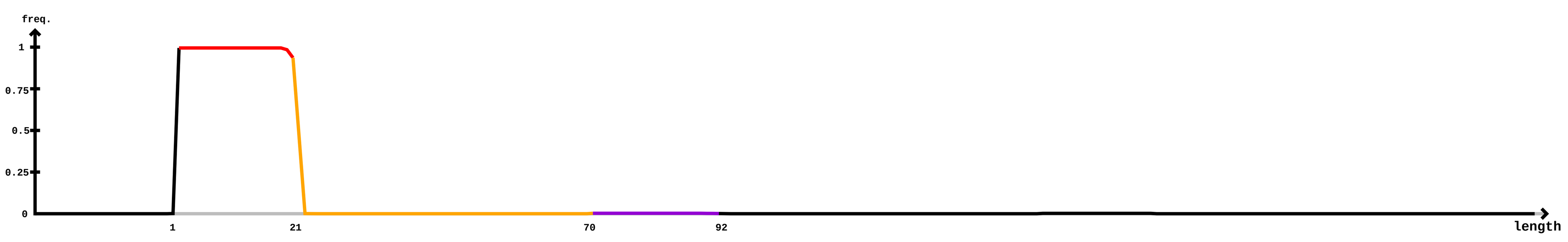


```
                      Mature                                                                Star
5'- uaguugggugagugguucuuguuggagaagcagggcacgugcaacuucaagccccaaaccccucucuuuggggcuagccaguagaauuuugcacgugcuccccuucuccaacauguguuucucacccucccugcagaagcauaaugaauuuuguacaaggauaugcuaaaugauaaugguaggaaauucugcuuucuuucacuaccuuauuccacauuauauaccaaacucuuguacaucuaucuauau   -3'   obs
    uaguugggugagugguucuuguuggagaagcagggcacgugcaacuucaagccccaaaccccucucuuuggggcuagccaguagaauuuugcacgugcuccccuucuccaacauguguuucucacccucccugcagaagcauaaugaauuuuguacaaggauaugcuaaaugauaaugguaggaaauucugcuuucuuucacuaccuuauuccacauuauauaccaaacucuuguacaucuaucuauau         exp
    ..(..((((((.....(.((((((((((..(((((((((((((...((((((((........)))))))))....))).......)))))))))))..))))))))))).).....)))))))..).(((....)))..........((((((((((((((...((((((((.((((((((..........)))).)))))))))....)))).)))))))......)))))))..........   reads   mm   sample
    .....................Cuggagaagcagggcacgu.................................................................................................................................................................................................................   1   1   LT-1
    .....................Cuggagaagcagggcacgugc...............................................................................................................................................................................................................   3   1   JCK-2
    .....................uuggagaagcagggcacgugc...............................................................................................................................................................................................................   1   0   LT-1
    .....................Cuggagaagcagggcacgugc...............................................................................................................................................................................................................   4   1   JCK-3
    .....................Cuggagaagcagggcacgugc...............................................................................................................................................................................................................   1   1   LT-2
    .....................uuggagaagcagggcacgugc...............................................................................................................................................................................................................   3   0   JCK-2
    .....................uuggagaagcagggcacgugc...............................................................................................................................................................................................................   3   0   LT-3
    .....................uuggagaagcagggcacgugc...............................................................................................................................................................................................................   1   0   JCK-3
    .....................Cuggagaagcagggcacgugca..............................................................................................................................................................................................................   1   1   LT-1
    .....................Cuggagaagcagggcacgugca..............................................................................................................................................................................................................   1   1   LT-3
    .....................Cuggagaagcagggcacgugca..............................................................................................................................................................................................................   3   1   JCK-3
    ......................uggagaagcagggcacgu.................................................................................................................................................................................................................   21   0   LT-2
    ......................uggagaagcagggcacgC.................................................................................................................................................................................................................   1   1   LT-3
    ......................uggagaagcagggcacgu.................................................................................................................................................................................................................   9   0   JCK-3
    ......................uggagaagcagggcacgu.................................................................................................................................................................................................................   33   0   LT-1
    ......................uggagaagcCgggcacgu.................................................................................................................................................................................................................   1   1   JCK-2
    ......................uggagaagcagggcacgu.................................................................................................................................................................................................................   34   0   LT-3
    ......................uggagaagcagggcacgu.................................................................................................................................................................................................................   31   0   JT-2
    ......................uggagaagcagggcacgu.................................................................................................................................................................................................................   26   0   JT-3
    ......................uggagaagcagggcacgu.................................................................................................................................................................................................................   2   0   JCK-1
    ......................uggagaagcagggcacgu.................................................................................................................................................................................................................   16   0   JCK-2
    ......................uggagaagcagggcacgu.................................................................................................................................................................................................................   19   0   LCK-3
    ......................uggagaagcagggcacgug................................................................................................................................................................................................................   49   0   LCK-3
    ......................uggCgaagcagggcacgug................................................................................................................................................................................................................   1   1   LT-2
    ......................uggagaagcagggcacguA................................................................................................................................................................................................................   2   1   JT-3
    ......................uggagaagcagggcaAgug................................................................................................................................................................................................................   1   1   LT-2
    ......................uggagaagUagggcacgug................................................................................................................................................................................................................   1   1   JCK-2
    ......................uggagaagcCgggcacgug................................................................................................................................................................................................................   1   1   LT-2
    ......................uggagaagcagggcacgug................................................................................................................................................................................................................   76   0   JT-3
    ......................uggagaagcagggcacgug................................................................................................................................................................................................................   122   0   LCK-2
    ......................uggagaagcagggcacgug................................................................................................................................................................................................................   54   0   JT-2
    ......................uggagaagcagggcacgug................................................................................................................................................................................................................   66   0   LT-1
    ......................uggagaagcagggcacgug................................................................................................................................................................................................................   122   0   LT-3
```

Mature Star

uaguugggugagugguucuuguuggagaagcagggcacgugcaacuucaagccccaaaccccucucuuuggggcuagccaguagaauuuugcacgugcuccccuucuccaacaugucuuucucacccucccugcagaagcauaaugaauuuuguacaaggauaugcuaaaugauaaugguaggaaauucugcuuucuuucacuaccuuauuccacauuauauauccaaacucuuguacaucuaucuauau

......................uggagaagUagggcacgug......	1	1	LT-1
......................uggagaagcagggNacgug......	1	1	LCK-1
......................uggagaagcaUggcacgug......	1	1	JT-3
......................uggagaagcagggcacgug......	53	0	JCK-2
......................uggCgaagcagggcacgug......	1	1	LT-3
......................uggagaagcagggcacgug......	109	0	LT-2
......................uggagaagcagggcacgug......	37	0	JCK-1
......................uggagaagcagggcacguA......	1	1	LT-3
......................uggagaagcagggcacgug......	127	0	LCK-1
......................uggagaagcagggcacgug......	41	0	JCK-3
......................uggagCagcagggcacgug......	1	1	LT-1
......................uggagaagcagggcacguC......	3	1	LT-3
......................uggagCagcagggcacgug......	1	1	JT-3
......................ugAagaagcagggcacgug......	1	1	LT-2
......................uggNgaagcagggcacgug......	1	1	JCK-1
......................uggagaagcagggcacguA......	7	1	LT-2
......................uggagaagUagggcacgugc......	10	1	JCK-2
......................uggCgaagcagggcacgugc......	4	1	LT-3
......................uggagaagcagggcaUgugc......	2	1	LT-3
......................uggaAaagcagggcacgugc......	1	1	JT-3
......................uggagaagcagggcacgugN......	1	1	JCK-1
......................uggagaagcagggcCcgugc......	1	1	JT-2
......................uggagaagcagggcacgugU......	2	1	JT-3
......................uggagaagcagggcCcgugc......	1	1	LT-1
......................ugAagaagcagggcacgugc......	2	1	LT-2
......................uggagaagcagggcacgugc......	604	0	JT-2
......................uAgagaagcagggcacgugc......	1	1	JCK-3
......................uggagaagUagggcacgugc......	2	1	JCK-1
......................uNgagaagcagggcacgugc......	1	1	JCK-3
......................uggagaCgcagggcacgugc......	1	1	JT-1
......................uggagaagcagggcacgugU......	21	1	JCK-2
......................Cggagaagcagggcacgugc......	2	1	LT-2
......................uggNgaagcagggcacgugc......	1	1	JCK-1
......................uggagaagUagggcacgugc......	2	1	JT-3
......................ugNagaagcagggcacgugc......	3	1	LT-2
......................uggagaagcagggcaUgugc......	1	1	JCK-1
......................uggagaagcagggcacgugc......	1129	0	JCK-3
......................uggagaagUagggcacgugc......	3	1	LT-3
......................ugNagaagcagggcacgugc......	1	1	LCK-1
......................uggCgaagcagggcacgugc......	1	1	JCK-3
......................uggagaagcagggcacguAc......	1	1	JCK-2
......................uggagCagcagggcacgugc......	2	1	JCK-1
......................uggagaagcagggcaUgugc......	2	1	JCK-2
......................uNgagaagcagggcacgugc......	1	1	LT-1
......................uggagaagcagNgcacgugc......	2	1	JCK-1
......................ugAagaagcagggcacgugc......	1	1	JCK-3
......................uggagaagcagggcNcgugc......	2	1	LCK-1
......................Cggagaagcagggcacgugc......	1	1	LT-3
......................uAgagaagcagggcacgugc......	4	1	LT-2
......................uggagaagcagggUacgugc......	1	1	JCK-3
......................uggagaagcagggcacgugc......	2380	0	LT-3
......................uggagaagcagggcGcgugc......	1	1	LCK-1
......................uggagaagcagggcacgugU......	11	1	JCK-3
......................uggagaagcagggNacgugc......	1	1	JT-2
......................uggagaagUagggcacgugc......	3	1	LT-2
......................uggagaagcCgggcacgugc......	1	1	JCK-3
......................uggCgaagcagggcacgugc......	1	1	LT-1
......................uggagaagcagggcacgugU......	4	1	JCK-1
......................uggagaagcCgggcacgugc......	1	1	JCK-1
......................uggagaagUagggcacgugc......	7	1	JCK-3
......................uggagCagcagggcacgugc......	4	1	LT-3
......................uggGgaagcagggcacgugc......	1	1	LCK-3
......................uggagaCgcagggcacgugc......	3	1	LT-2
......................uggCgaagcagggcacgugc......	1	1	LCK-1
......................uggagaCgcagggcacgugc......	3	1	JCK-3
......................uggagaagcagggcacgugc......	1369	0	LT-2
......................ugAagaagcagggcacgugc......	1	1	LCK-3
......................uggagaagcCgggcacgugc......	3	1	LT-2
......................uggagaagcagggcCcgugc......	1	1	JCK-3
......................uggagaagcagggcacgugc......	318	0	LCK-2

```
                      Mature                                                               Star
uaguugggugagugguucuuguuggagaagcagggcacgugcaacuucaagccccaaaccccucucuuuggggcuagccaguagaauuuugcacgugcuccccuucuccaacauguguuucucacccucccugcagaagcauaaugaauuuuguacaaggauaugcuaaaugauaaugguaggaaauucugcuuucuuucacuaccuuauuccacauuauauauccaacucuuguacaucuaucuauau
......................uggagCagcagggcacgugc............................................................................................................................................................................................... 1     1   LT-2
......................uggagaagcagggcacgugc............................................................................................................................................................................................... 319   0   LCK-1
......................uggaAaagcagggcacgugc............................................................................................................................................................................................... 1     1   LT-3
......................uggagaagAagggcacgugc............................................................................................................................................................................................... 1     1   JCK-2
......................uggCgaagcagggcacgugc............................................................................................................................................................................................... 1     1   JCK-1
......................uggagaCgcagggcacgugc............................................................................................................................................................................................... 6     1   JCK-2
......................uNgagaagcagggcacgugc............................................................................................................................................................................................... 1     1   LCK-1
......................uUgagaagcagggcacgugc............................................................................................................................................................................................... 1     1   JCK-3
......................uggagaCgcagggcacgugc............................................................................................................................................................................................... 7     1   LT-3
......................uggCgaagcagggcacgugc............................................................................................................................................................................................... 11    1   LT-2
......................uggagaagcaggAcacgugc............................................................................................................................................................................................... 1     1   LCK-3
......................uggagaagcagggcCcgugc............................................................................................................................................................................................... 1     1   LCK-2
......................uggagGagcagggcacgugc............................................................................................................................................................................................... 1     1   LT-1
......................uggagaCgcagggcacgugc............................................................................................................................................................................................... 1     1   LCK-2
......................uggagaagcagggcCcgugc............................................................................................................................................................................................... 2     1   LT-2
......................uggagaagcagggcCcgugc............................................................................................................................................................................................... 3     1   JCK-2
......................uggagaagUagggcacgugc............................................................................................................................................................................................... 1     1   LT-1
......................uggagaagcagggcacgugc............................................................................................................................................................................................... 532   0   LCK-3
......................uggagaagcagggcacgCgc............................................................................................................................................................................................... 1     1   LT-3
......................uggagaagcagggcacgugU............................................................................................................................................................................................... 16    1   LT-3
......................uggagaagcagggcCcgugc............................................................................................................................................................................................... 1     1   JT-3
......................uggagaCgcagggcacgugc............................................................................................................................................................................................... 2     1   LT-1
......................uggCgaagcagggcacgugc............................................................................................................................................................................................... 1     1   JT-2
......................uAgagaagcagggcacgugc............................................................................................................................................................................................... 1     1   JCK-1
......................uggagaagcCgggcacgugc............................................................................................................................................................................................... 1     1   LCK-3
......................uggagGagcagggcacgugc............................................................................................................................................................................................... 1     1   LCK-1
......................uggagaagcagggcacgugU............................................................................................................................................................................................... 11    1   LCK-3
......................uggagCagcagggcacgugc............................................................................................................................................................................................... 2     1   JCK-2
......................uggagaagcagggcacgugc............................................................................................................................................................................................... 2130  0   LT-1
......................uggagaCgcagggcacgugc............................................................................................................................................................................................... 1     1   JT-2
......................uggNgaagcagggcacgugc............................................................................................................................................................................................... 2     1   JT-2
......................uggagaagcagggcacgugU............................................................................................................................................................................................... 44    1   LT-2
......................uggagCagcagggcacgugc............................................................................................................................................................................................... 1     1   JCK-3
......................uggagaagcCgggcacgugc............................................................................................................................................................................................... 1     1   JCK-2
......................uggagaagcagggcacgugc............................................................................................................................................................................................... 1625  0   JCK-2
......................Cggagaagcagggcacgugc............................................................................................................................................................................................... 1     1   JCK-3
......................uggCgaagcagggcacgugc............................................................................................................................................................................................... 3     1   JCK-2
......................uggagaagcGgggcacgugc............................................................................................................................................................................................... 2     1   LT-3
......................uggagaagcCgggcacgugc............................................................................................................................................................................................... 1     1   JT-2
......................uggagaagcagggNacgugc............................................................................................................................................................................................... 1     1   LCK-1
......................uggagaagcagggcCcgugc............................................................................................................................................................................................... 3     1   JCK-1
......................uggagaagcagggcacgugc............................................................................................................................................................................................... 121   0   JT-1
......................uggagaagcCgggcacgugc............................................................................................................................................................................................... 5     1   LT-3
......................uNgagaagcagggcacgugc............................................................................................................................................................................................... 2     1   JCK-2
......................uggagaagcCgggcacgugc............................................................................................................................................................................................... 1     1   LT-1
......................uggagaagcagggcacgugU............................................................................................................................................................................................... 25    1   LT-1
......................uggagaagcagggcacgugc............................................................................................................................................................................................... 875   0   JCK-1
......................uggagaagcagggcacgugc............................................................................................................................................................................................... 857   0   JT-3
......................uggagaagcagAgcacgugc............................................................................................................................................................................................... 1     1   LT-3
......................Gggagaagcagggcacgugc............................................................................................................................................................................................... 1     1   LCK-2
......................uggagaagcagggcCcgugc............................................................................................................................................................................................... 3     1   LT-3
......................uAgagaagcagggcacgugc............................................................................................................................................................................................... 1     1   LT-1
......................uggagCagcagggcacgugc............................................................................................................................................................................................... 3     1   LT-1
......................uggagaagcagggcaAgugca.............................................................................................................................................................................................. 1     1   JCK-1
......................uggagaagcagggcacgugca.............................................................................................................................................................................................. 1285  0   JCK-2
......................uggagaagcagggcacgugcU.............................................................................................................................................................................................. 7     1   JCK-2
......................uggagaagcagggcaGgugca.............................................................................................................................................................................................. 1     1   JCK-2
......................uggagaagcagggcacAugca.............................................................................................................................................................................................. 1     1   JT-3
......................uggagaCgcagggcacgugca.............................................................................................................................................................................................. 1     1   JCK-1
......................uggagaagcagggcacgugcC.............................................................................................................................................................................................. 7     1   LT-1
......................uggagaagUagggcacgugca.............................................................................................................................................................................................. 1     1   LT-1
......................uggagaagcagggcacgugcC.............................................................................................................................................................................................. 4     1   LT-2
......................uggagaagcagggcacgugUa.............................................................................................................................................................................................. 1     1   LT-3
......................uggagaagcagggcacgugcC.............................................................................................................................................................................................. 9     1   JCK-2
......................uggagaagcagggcacgugca.............................................................................................................................................................................................. 360   0   LT-1
......................uggagaCgcagggcacgugca.............................................................................................................................................................................................. 1     1   JCK-3
......................uggagCagcagggcacgugca.............................................................................................................................................................................................. 1     1   LT-2
......................uggagaagcagggcacgugcC.............................................................................................................................................................................................. 4     1   JCK-3
......................uggagaagcagNgcacgugca.............................................................................................................................................................................................. 2     1   JCK-1
......................uggagaagcagggcacgugca.............................................................................................................................................................................................. 805   0   JCK-1
```

```
                      Mature                                                                             Star
uaguugggugagugguucuuguuggagaagcagggcacgugcaacuucaagccccaaaccccucucuuuggggcuagccaguagaauuuugcacgugcuccccuucuccaacauguguuucucacccucccugcagaagcauaaugaauuuuguacaaggauaugcuaaaugauaaugguaggaaauucugcuuucuuucacuaccuuauuccacauuauauauccaaacucuuguacaucuaucuauau
......................uggagaagcagggcacgugca...............................................................................................................................................................................   2     0     LCK-2
......................uNgagaagcagggcacgugca...............................................................................................................................................................................   1     1     JCK-1
......................uggNgaagcagggcacgugca...............................................................................................................................................................................   1     1     JCK-1
......................uggCgaagcagggcacgugca...............................................................................................................................................................................   1     1     JCK-1
......................uggagaagcagggcaUgugca...............................................................................................................................................................................   1     1     JCK-2
......................uggagaagcagggcacAugca...............................................................................................................................................................................   2     1     JT-2
......................uggagaCgcagggcacgugca...............................................................................................................................................................................   3     1     JCK-2
......................uggagaagcagggcacgugca...............................................................................................................................................................................   100   0     JT-1
......................uggagaagcagggcCcgugca...............................................................................................................................................................................   1     1     JCK-3
......................uggagaagUagggcacgugca...............................................................................................................................................................................   6     1     JCK-3
......................uNgagaagcagggcacgugca...............................................................................................................................................................................   1     1     JCK-3
......................uggagCagcagggcacgugca...............................................................................................................................................................................   1     1     JCK-3
......................uggagCagcagggcacgugca...............................................................................................................................................................................   1     1     JCK-2
......................uAgagaagcagggcacgugca...............................................................................................................................................................................   1     1     JCK-3
......................uggagaagcagggcaGgugca...............................................................................................................................................................................   1     1     JCK-1
......................uggagaagcagggcacgugca...............................................................................................................................................................................   143   0     LT-2
......................uggagaagUagggcacgugca...............................................................................................................................................................................   3     1     JCK-1
......................uggagaagcagggcacgugca...............................................................................................................................................................................   58    0     LCK-3
......................uggagaCgcagggcacgugca...............................................................................................................................................................................   1     1     LT-3
......................uggagaagcagggcacgugca...............................................................................................................................................................................   131   0     JT-3
......................uggCgaagcagggcacgugca...............................................................................................................................................................................   1     1     JCK-2
......................uggagaagcagggcCcgugca...............................................................................................................................................................................   1     1     LT-2
......................uggagaagcagggcCcgugca...............................................................................................................................................................................   1     1     LT-1
......................uggagaagcagggUacgugca...............................................................................................................................................................................   1     1     LT-1
......................uggagaCgcagggcacgugca...............................................................................................................................................................................   1     1     JT-3
......................uggagaagcagggcacgugcC...............................................................................................................................................................................   3     1     LT-3
......................uggagaagcagggcacgugNa...............................................................................................................................................................................   1     1     JCK-1
......................uggCgaagcagggcacgugca...............................................................................................................................................................................   2     1     LT-1
......................uggagaagcagggcGcgugca...............................................................................................................................................................................   1     1     JCK-3
......................uggagaagcagggcacgugcU...............................................................................................................................................................................   13    1     LT-1
......................uggagaagcagggcacgugcU...............................................................................................................................................................................   6     1     LT-3
......................uggagaagcagggcacgugcU...............................................................................................................................................................................   16    1     JT-1
......................uNgagaagcagggcacgugca...............................................................................................................................................................................   5     1     JCK-2
......................uggagaagcagggcacgugUa...............................................................................................................................................................................   4     1     LT-2
......................uggagaagcagggcacgugca...............................................................................................................................................................................   317   0     LT-3
......................uggagaagcagggcacgugUa...............................................................................................................................................................................   1     1     LT-1
......................uggagaagcagggcacgugca...............................................................................................................................................................................   92    0     JT-2
......................uggagaagcagggcacgugcU...............................................................................................................................................................................   19    1     LCK-3
......................uggagaagUagggcacgugca...............................................................................................................................................................................   6     1     JCK-2
......................uggagaagUagggcacgugca...............................................................................................................................................................................   1     1     JT-1
......................uggagaagcagggcacgugcU...............................................................................................................................................................................   1     1     JCK-1
......................uggagaagcagggcacgugcC...............................................................................................................................................................................   2     1     JCK-1
......................uggagaagcCgggcacgugca...............................................................................................................................................................................   1     1     JCK-1
......................uggagaagcagggcacgugcG...............................................................................................................................................................................   1     1     JCK-2
......................uggagaagcagggcacgugca...............................................................................................................................................................................   1008  0     JCK-3
......................uggagaagcagggcacgugcU...............................................................................................................................................................................   10    1     LT-2
......................uggagaagcagggcCcgugca...............................................................................................................................................................................   1     1     JCK-2
......................uggagCagcagggcacgugca...............................................................................................................................................................................   1     1     JCK-1
......................uggagaagcagggcacgugcaa..............................................................................................................................................................................   1     0     JCK-1
......................uggagaagcagggcacgugcaa..............................................................................................................................................................................   3     0     JCK-2
......................uggagaagcagggcacgugcaa..............................................................................................................................................................................   3     0     JCK-3
......................uggagaagcagggcacgugcaa..............................................................................................................................................................................   1     0     LT-1
......................uggagaagcagggcacgugcaC..............................................................................................................................................................................   1     1     JCK-1
......................uggagaagcagggcacgugcaG..............................................................................................................................................................................   1     1     LT-1
......................uggagaagcagggcacgugcaac.............................................................................................................................................................................   2     0     JCK-2
......................uggagaagcagggcacgugcaUc.............................................................................................................................................................................   3     1     JCK-3
......................uggagaagcagggcacgugcaac.............................................................................................................................................................................   1     0     LT-2
......................uggagaagcagggcacgugcaaUu............................................................................................................................................................................   1     1     LT-3
........................gagaagcagggcacgugc................................................................................................................................................................................   2     0     JCK-3
...........................................................................................cacgugcuccccuucucc.............................................................................................................................................   1     0     JCK-1
...........................................................................................cacgugcuccccuucucca............................................................................................................................................   6     0     LCK-3
...........................................................................................cacgugcuccccuucucca............................................................................................................................................   3     0     JCK-2
...........................................................................................cacgugcuccccuucucca............................................................................................................................................   2     0     JCK-1
...........................................................................................cacgugcuccccuucuccaa...........................................................................................................................................   2     0     LT-3
...........................................................................................cacgugcuccccuucuccaac..........................................................................................................................................   3     0     JCK-3
...........................................................................................cacgugcucUccuucuccaac..........................................................................................................................................   1     1     LT-3
...........................................................................................cacgugcuccccuucuccaac..........................................................................................................................................   2     0     JCK-1
...........................................................................................cacgugcuccccuucuccaac..........................................................................................................................................   2     0     LT-1
...........................................................................................cacgugcuccccuucuccaac..........................................................................................................................................   5     0     JCK-2
...........................................................................................cacgugcuccccuucuccaaA..........................................................................................................................................   1     1     LT-1
```

```
                    Mature                                                         Star
uaguugggugagugguucuuguuggagaagcagggcacgugcaacuucaagccccaaaccccucucuuuggggcuagccaguagaauuuugcacgugcuccccuucuccaacauguguuucucacccucccugcagaagcauaaugaauuuuguacaaggauaugcuaaaugauaaugguaggaaauucugcuuucuuucacuaccuuauuccacauuauauauccaaacucuuguacaucuaucuauau
...........................................................................................cacgugcuccccuucuccaacU.........................................................................................................................................   2   1   JCK-3
...........................................................................................cacgugcuccccuucuccaacU.........................................................................................................................................   8   1   JCK-2
...........................................................................................cacgugcuccccuucuccaacU.........................................................................................................................................   3   1   LT-1
...........................................................................................cacgugcuccccuucuccaacU.........................................................................................................................................   3   1   LT-2
...........................................................................................cacgugcuccccuucuccaacU.........................................................................................................................................   5   1   JCK-1
............................................................................................acgugcuccccuucucca............................................................................................................................................   1   0   JCK-2
................................................................................................................................................uaaaugauaauAguaggaa.......................................................................................   18  1   LCK-1
................................................................................................................................................uaaaugauaauAguaggaa.......................................................................................   21  1   LCK-2
................................................................................................................................................uaaaugauaauCguaggaa.......................................................................................   1   1   LCK-2
```
